# Efflux pumps as potential targets for biofilm inhibition

**DOI:** 10.3389/fmicb.2024.1315238

**Published:** 2024-02-23

**Authors:** Jingyi Ren, Meijuan Wang, Wenjuan Zhou, Zhonghao Liu

**Affiliations:** Department of Implantology, Yantai Stomatological Hospital Affiliated to Binzhou Medical University, Yantai, China

**Keywords:** efflux pump, biofilm, motility, quorum sensing, efflux pump inhibitor

## Abstract

Biofilms account for a great deal of infectious diseases and contribute significantly to antimicrobial resistance. Efflux pumps confer antimicrobial resistance to microorganisms and involve multiple processes of biofilm formation. Efflux pump inhibitors (EPIs) are attracting considerable attention as a biofilm inhibition strategy. The regulatory functions of efflux pumps in biofilm formation such as mediating adherence, quorum sensing (QS) systems, and the expression of biofilm-associated genes have been increasingly identified. The versatile properties confer efflux pumps both positive and negative effects on biofilm formation. Furthermore, the expression and function of efflux pumps in biofilm formation are species-specific. Therefore, this review aims to detail the double-edged sword role of efflux pumps in biofilm formation to provide potential inhibition targets and give an overview of the effects of EPIs on biofilm formation.

## 1 Introduction

Biofilms are communities of microorganisms embedded within self-produced extracellular polymeric substances (EPSs) composed of lipids, amyloids, polysaccharides, extracellular DNA, and proteins (Flemming et al., 2023). Biofilms protect microorganisms from unfavorable environments such as altered pH, osmolarity, nutrient restriction, and mechanical/shear forces (Sun et al., 2013; Bjarnsholt et al., 2018). The distinct properties of biofilms including the impermeability, low growth rates, overexpression of efflux pumps, and the existence of persister cells enhance antimicrobial resistance greatly (Sharma et al., 2019). Furthermore, biofilms also provide an ideal niche for horizontal transfer of antimicrobial resistance genes (Mah, 2012). Hence, biofilms impose more challenges to the dissemination of antimicrobial resistance.

Approximately 80% of infectious diseases including lung infections, urinary tract infections, endocarditis, rhinosinusitis, prostatitis, periodontitis, and caries are caused by biofilms (Burmølle et al., 2010). Especially the treatment for medical device-related infections has been problematic. Medical device-related infections are caused by the biofilms present on medical implants such as orthopedic/dental implants, intravascular/urinary catheters, and vascular prostheses (Costerton et al., 2005). These infections are not treatable by conventional antimicrobial therapy. As a consequence, the removal of infected medical implants is usually required to eradicate infections (Veerachamy et al., 2014). The failure of medical implants and mortality attributed to biofilm formation are continuing concerns.

Efflux pumps are membrane proteins encoded by genes located in both chromosomes and plasmids. Efflux pumps widely exist in all bacterial species and are able to expel a wide spectrum of substrates including antibiotics, biocides, dyes, heavy metals, toxins, and metabolites out of cells. Thus, the overexpression of efflux pumps leads to antimicrobial resistance by impeding the intracellular accumulation. To date, multidrug efflux pumps are categorized into six families based on topology, structure, and energetics: the ATP-binding cassette (ABC) superfamily, the major facilitator superfamily (MFS), the resistance-nodulation–cell-division (RND) superfamily, the small multidrug resistance (SMR) family, the multidrug and toxic compound extrusion (MATE) family, and the proteobacterial antimicrobial compound efflux (PACE) family (Du et al., 2018). Even though hundreds of bacterial efflux pumps have been characterized, a myriad of efflux pumps still remain undiscovered.

In addition to antimicrobial resistance, a growing body of literature has recognized the involvement of efflux pumps in diverse phases of biofilm formation in recent years. The discovery of decreased biofilm formation caused by the loss of efflux pumps sheds new light on EPIs as biofilm control strategies. Thus, this review summarizes the evidence concerning the regulatory effects of efflux pumps and EPIs on biofilm formation.

## 2 Biofilm formation

**Figure 1:**
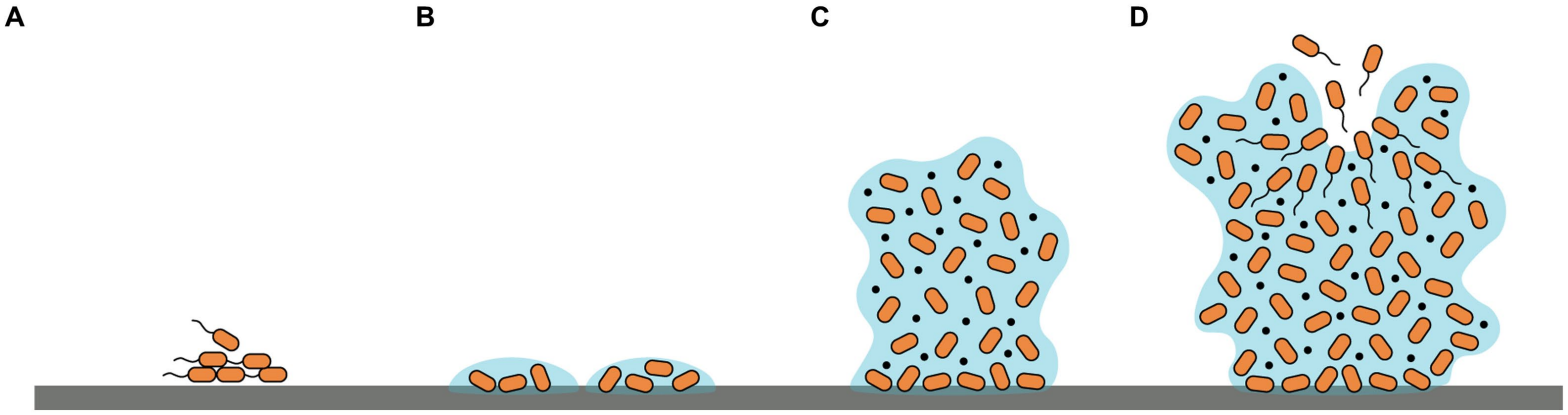
Schematic representation of the four phases of biofilm formation: **(A)** reversible adherence to surfaces; **(B)** irreversible adherence and formation of microcolonies; **(C)** proliferation of cells and production of EPSs establishing a three-dimensional structure; **(D)** dispersal of planktonic cells to re-build new biofilms.

Biofilm formation is a complicated process involving multiple signal transduction pathways. Briefly, four phases are included: (i) reversible attachment of microbes to a surface or each other; (ii) irreversible attachment to the surface and formation of microcolonies; (iii) proliferation of cells and synthesis of EPSs establishing a multilayer three-dimensional structure; (iv) detachment and dispersal of planktonic cells from mature biofilms to re-build new biofilms (Tolker-Nielsen, 2015; Armbruster and Parsek, 2018) (Figure 1). The initial phase of biofilm formation could be influenced by diverse factors such as bacterial species, environmental conditions, surface characters as well as gene products (Pandey et al., 2022). Additionally, microorganism motilities also play a crucial role by facilitating sensing and contacting favorable adhered surfaces, translocation, and reversible/irreversible adherence of bacterial cells to surfaces (Khan et al., 2020). Conversely, during the growth and mature phase of biofilm formation, the inhibition of motilities aids the maintenance of bacterial aggregation (Guttenplan and Kearns, 2013). Predominant motilities include swimming, swarming, gliding, and twitching (Harshey, 2003). Swimming and swarming motilities are largely driven by flagella, rotation, and chemotaxis while twitching motility relies on type IV pili (Wadhwa and Berg, 2022).

Additionally, QS systems play a pivotal role in biofilm development. QS is a communication mechanism between cells to cells in response to environmental signals to modulate cell density (Ge et al., 2022). The signal molecules are called autoinducers (AIs). The generation, diffusion, and exportation of AIs increase concomitantly with the rise of cell population. Once the concentration of AIs reaches a certain threshold, the AIs initiate binding to cognate receptors triggering a coordinated shift of gene expression, which promotes biofilm formation and relative virulence (Antonioli et al., 2018; Giannakara and Koumandou, 2022). The AIs of Gram-positive bacteria are usually oligopeptides (autoinducing peptides, AIPs), while the AIs of Gram-negative bacteria are acyl-homoserine lactones (AHLs) or S-adenosyl methionine (SAM)-products (Papenfort and Bassler, 2016).

## 3 The effects of efflux pumps on biofilm formation

Possible mechanisms by which efflux pumps impact biofilm formation mainly include: (i) impacting initial adherence; (ii) transporting metabolites and QS system signals; (iii) extruding harmful substances; (iv) indirectly mediating biofilm-associated genes. Diverse functions confer a double-edged sword role to efflux pumps in different stages of biofilm formation. On the one hand, the expression of some efflux pumps contributes to biofilm formation by promoting the initial adherence of microorganisms and/or the production of biofilm-associated extracellular matrix (Baugh et al., 2012; Richmond et al., 2016; Knight et al., 2018). On the other hand, the expression of some efflux pumps has been recognized to alleviate biofilm formation by mediating AIs of QS systems and biofilm-associated gene expression (Lamarche and Déziel, 2011; Zhu et al., 2011). Although the overexpression of various efflux pumps has been identified in biofilms compared to planktic cells, the efflux pumps that can cause definitive impacts on biofilm formation are less evident. For example, the MdtJ efflux pump (SMR-type), responsible for exporting spermidine, plays a role in the stimulation and promotion of biofilm formation in *Escherichia coli*. Nevertheless, no alterations in intracellular spermidine concentration and biofilm formation were detected in the *mdtJ* deletion mutation (Thongbhubate et al., 2021). In this regard, the MdtJ efflux pump is not a promising target of EPIs for biofilm inhibition in *E. coli*. Therefore, to pave the way for selecting EPI targets, herein the evidence regarding the efflux pumps with definitive impacts on biofilm formation will be reviewed (Table 1).

**Table 1:** Effects of efflux pumps on biofilm formation.

Effects on biofilm formation	Efflux pump type	Efflux pump	Effects of deletion or overexpression on biofilm formation	Species	References
**Positive effects**	**RND**	AdeB	Reduced by deletion	*Acinetobacter baumannii*	Richmond et al. (2016)
					Yoon et al. (2015)
		AdeJ	Reduced by deletion	*Acinetobacter nosocomialis*	Knight et al. (2018)
		AcrB and Mdt ABC	Reduced by deletion	*Escherichia coli*	Yamasaki et al. (2015)
		AcrB AcrD AcrEF MdtABC MdsABD	Reduced by deletion	*Salmonella enterica* serovar Typhimurium	Baugh et al. (2012)
					Baugh et al. (2014)
		AcrD AcrE MdtE	Reduced by deletion	*Escherichia coli*	Matsumura et al. (2011)
		AcrA and AcrB	Enhanced by overexpression	*Acinetobacter nosocomialis*	Subhadra et al. (2018)
		SmeYZ	Reduced by deletion	*Stenotrophomonas maltophilia*	Lin et al. (2015)
		AreABC AreDEF AreGHI	Reduced by deletion	*Aliarcobacter butzleri*	Mateus et al. (2021)
		AmrAB-OprA	Reduced by deletion	*Burkholderia pseudomallei*	Mima and Schweizer (2010)
		BpeAB	Reduced by deletion	*Burkholderia pseudomallei*	Chan and Chua (2005)
		A1s_0116	Reduced by deletion	*Acinetobacter baumannii*	Blaschke et al. (2021)
	**MFS**	NorA	Enhanced by overexpression	*Staphylococcus pseudintermedius*	Rampacci et al. (2022)
		Bcr	Reduced by disruption	*Proteus mirabilis*	Holling et al. (2014)
		EmrA EmrB	Reduced by deletion	*Acinetobacter baumannii*	Lin et al. (2020)
		EmrAB MdfA	Reduced by deletion	*Salmonella enterica* serovar Typhimurium	Baugh et al. (2012)
		EmrD EmrK	Reduced by deletion	*Escherichia coli*	Matsumura et al. (2011)
		AbaF	Reduced by deletion	*Acinetobacter baumannii*	Sharma et al. (2017)
		Rv1877	Enhanced by expression	*Escherichia coli*	Adhikary et al. (2022)
	**ABC**	MacAB-TolC	Reduced by deletion	*Acinetobacter baumannii*	Robin et al. (2021)
		MacAB	Reduced by deletion	*Salmonella enterica* serovar Typhimurium	Baugh et al. (2012)
	**SMR**	EmrE	Reduced by deletion	*Escherichia coli*	Matsumura et al. (2011)
	**MATE**	MdtK	Reduced by deletion	*Salmonella enterica* serovar Typhimurium	Baugh et al. (2012)
					Nishino et al. (2006)
**Negative effects**	**RND**	AcrE	Enhanced by deletion	*Escherichia coli*	Bay et al. (2017)
		MexEF-OprN	Reduced by overexpression	*Pseudomonas aeruginosa*	Favre-Bonté et al. (2003)
					Lamarche and Déziel (2011)
		AdeABC AdeIJK	Reduced by overexpression	*Acinetobacter baumannii*	Yoon et al. (2015)
	**MFS**	EmrA	Enhanced by deletion	*Escherichia coli*	Bay et al. (2017)
	**MATE**	MdtK	Enhanced by deletion	*Escherichia coli*	Bay et al. (2017)
	**ABC**	lm.G_1771	Enhanced by inactivation	*Listeria monocytogenes*	Zhu et al. (2008)

### 3.1 Positive effects

Previous research has recognized an array of efflux pumps with positive effects on biofilm formation by finding attenuated biofilm formation caused by the lack of these efflux pumps. In this context, these efflux pumps can be considered as potential EPI targets for biofilm inhibition. Generally, the positive effects of efflux pumps mainly rely on mediating microorganism motilities and the production of biofilm-associated extracellular matrix. Thus, the absence of these efflux pumps can compromise the adherence of microorganisms and/or the establishment of mature biofilm structures thereby leading to diminished biofilm formation. However, the mechanisms underlying the positive effects of many efflux pumps on biofilm formation have not yet been clearly elucidated.

#### 3.1.1 RND-type efflux pumps

The RND-type efflux pumps are ubiquitous in all domains of life and are mainly responsible for antimicrobial resistance in Gram-negative bacteria. Well-known RND-type efflux pumps include the AcrAB-TolC efflux system and related Acr pumps in *E. coli*, Mex efflux systems in *Pseudomonas* sp., and Ade efflux systems in *Acinetobacter* sp. (Du et al., 2018).

##### 3.1.1.1 Ade efflux systems

The AdeABC and AdeIJK efflux pumps belonging to Ade efflux systems are closely associated with antimicrobial resistance in *Acinetobacter baumannii* (Abd El-Rahman et al., 2023). It has been demonstrated that the deletion of *adeB* decreased biofilm formation in *A. baumannii* (Yoon et al., 2015; Richmond et al., 2016). Consistently, a significant downregulation of type IV pilus genes which affect natural transformation, twitching motility, and biofilm formation was observed in the *adeB* deletion mutant (Richmond et al., 2016). It is noteworthy that the lack of *adeB* had no influence on the number of adherent cells on the mucosal tissue, but the inhibition of mature biofilm establishment was found by confocal microscopy. These results indicate the positive effect of *adeB* on the mature phase of biofilm formation.

Nevertheless, the expression of *adeB* in *A. baumannii* clinical isolates has been conflicting. Previous research failed to detect the association between the expression of *adeB* and biofilm formation in *A. baumannii* clinical isolates (Chen et al., 2020; Abd El-Rahman et al., 2023). Another study conducted by Kim et al. (2021) revealed a negative correlation between the expression level of *adeB* and biofilm formation capacity in *A. baumannii* clinical isolates (n = 120). However, Phe-Arg β-naphthylamide (PAβN), an EPI, significantly diminished biofilm formation suggesting the existence of other underlying efflux pumps related to biofilm formation in clinical isolates (Chen et al., 2020). Although there is little agreement on the association between the efflux pump expression and biofilm formation in *A. baumannii* clinical strains, the evidence together indicates the discrepancy in efflux pump expression among different strains of the same species.

In contrast to previous findings in *A. baumannii* (Yoon et al., 2015; Richmond et al., 2016), no alterations of biofilm formation were detected in the *ΔadeB* or *ΔadeB*/*adeJ* mutants of *Acinetobacter nosocomialis* (Knight et al., 2018). Nevertheless, the single deletion of *adeJ* led to decreased biofilm formation suggesting the positive effect of the AdeIJK efflux pump on biofilm formation in *A. nosocomialis* (Knight et al., 2018). Moreover, a remarkable reduction (over 50%) in surface motility was observed in the *ΔadeJ* mutant, while no changes in the secretion level of 3-OH C12-HSL, an AI of QS systems, were detected (Knight et al., 2018). These findings suggest that the impaired biofilm formation caused by the lack of *adeJ* was associated with reduced surface motility but independent of QS systems. Considering all of the evidence, it seems that the efflux pump expression related to biofilm formation is species- and strain-specific.

##### 3.1.1.2 AcrAB–TolC efflux system

The AcrAB–TolC efflux system is able to transport a wide spectrum of substrates and is associated with antimicrobial resistance in various species including *E. coli*, *Salmonella enterica* serovar Typhimurium (*Salmonella*), *Enterobacter aerogenes*, *Klebsiella pneumoniae*, and *Enterobacter cloacae* (Blair et al., 2015). It comprises three components including an inner membrane protein AcrB, a periplasmic adapter protein AcrA, and an outer membrane protein TolC (Koronakis et al., 2004). The effects of AcrAB on biofilm formation have been controversially documented. Baugh et al. (2012) explored the biofilm formation of multiple efflux mutants including RND mutants lacking *acrB*, *acrD*, *acrEF*, *mdtABC* or *mdsABD*, MFS mutants lacking *emrAB* or *mdfA*, a MATE mutant lacking *mdtK*, an ABC mutant lacking *macAB*, and a *tolC* mutant in *Salmonella*. All tested mutants showed reduced biofilm formation. The scanning electron microscopy results exhibited that the adherence to surfaces was not compromised, but mutants failed to form the three-dimensional structure of biofilm. Congo red assays confirmed the loss of the ability to produce curli. Consistently, the production of *csgB* and *csgD* genes involving curli biosynthesis was downregulated in all mutants. Curli are the crucial proteinaceous component of EPSs in various species such as *E. coli* and *Salmonella* spp. responsible for surface attachment and cell aggregation during biofilm formation (Barnhart and Chapman, 2006). These findings suggest the positive effects of efflux pumps on biofilm formation by modulating the production of biofilm-associated extracellular matrix. Later, the same team (Baugh et al., 2014) further elucidated that the lack of TolC or AcrB resulted in a more significant reduction in biofilm formation than lacking AcrA alone in *Salmonella*. In line with previous findings, the compromised biofilm formation resulted from impaired curli biosynthesis while independent of cellular hydrophobicity or aggregation. AcrD and AcrE are close homologues of AcrB and AcrA, respectively (Alav et al., 2021). The substrates of AcrE are similar to AcrAB while AcrD mainly contributes to the exporting of aminoglycosides and anionic β-lactams (Aires and Nikaido, 2005). The study in *E. coli* also supports earlier results in *Salmonella* by finding that the individual deletion of *acrD*, *acrE*, or *mdtE* significantly compromised biofilm formation (Matsumura et al., 2011).

However, these observations have been challenged by a study reporting no alterations of biofilm formation following the deletion of *acrB* or both *acrA* and *acrB* in *Salmonella* under normal growth conditions (Schlisselberg et al., 2015). The deletion of these efflux genes only attenuated biofilm formation under antimicrobial selective pressure (Schlisselberg et al., 2015). One possible explanation is that in efflux deletion mutants, other efflux pumps were exploited for the expelling of antimicrobials leading to a more severe deficiency of efflux pumps available for biofilm formation. The results address the impact of antimicrobial selective pressure on the expression of efflux pumps and indicate the potential of the combined application of EPIs and antimicrobials.

AcrR is a repressor of AcrAB in *A. nosocomialis* (Subhadra et al., 2018). In the *acrR* deletion mutant, the upregulation of *acrA* and *acrB* genes led to increased biofilm formation and invasion in *A. nosocomialis* (Subhadra et al., 2018). Furthermore, the upregulation of *acrA* and *acrB* genes enhanced motility and upregulated the expression of *csuC* and *csuD* genes (Subhadra et al., 2018). *CsuC* and *csuD* genes encode proteins of the CsuA/BABCDE chaperone-usher pili assembly system, which is important for the initial adhesion of biofilm formation (Kishii et al., 2020). This study revealed the role of AcrAB contributing to biofilm formation by promoting the initial adherence of biofilm formation. Conflictingly, the single deletion of *acrB* or *mdtABC* had no impact on biofilm formation in *E. coli* (Yamasaki et al., 2015). However, impaired biofilm formation was observed in the mutant lacking both *acrB* and *mdtABC* suggesting the synergistic function of AcrB and Mdt ABC efflux pumps on biofilm formation in *E. coli*. Additionally, the impact of these efflux pumps on biofilm formation is time-dependent. No alteration was found during the initial stage, but the maintenance of biofilm formation was impaired by the loss of efflux pumps (Yamasaki et al., 2015).

##### 3.1.1.3 SmeYZ efflux pump

SmeYZ, one of the putative RND-type efflux pumps in *Stenotrophomonas maltophilia*, is mainly related to multiple physiological functions but not antimicrobial resistance (Crossman et al., 2008; Gould et al., 2013). Lin et al. (2015) demonstrated the positive effect of SmeYZ on biofilm formation in *S. maltophilia*. The *smeYZ* deletion mutant failed to produce flagellar structures which are essential organelles for microorganism motilities thus leading to a defect of biofilm formation. Furthermore, reduced swimming and oxidative stress susceptibility were observed in the *smeYZ* deletion mutant during the initial adherence stage. The results imply the positive regulatory effect of SmeYZ on the early phase of biofilm formation.

##### 3.1.1.4 Are efflux systems

The AreABC, AreDEF, and AreGHI efflux pumps are putative efflux pumps in the genome of *Aliarcobacter butzleri* responsible for antimicrobial resistance especially against erythromycin (Isidro et al., 2020). Mateus et al. (2021) assessed the effects of AreABC, AreDEF, and AreGHI on the virulence of *A. butzleri*. Decreased biofilm formation was observed in all the efflux deletion mutants. Nevertheless, only the alleviated biofilm formation in the Δ*areG* mutant was found to be associated with reduced motility. The mechanisms of how the other two efflux pumps regulated biofilm formation remain unknown.

##### 3.1.1.5 AmrAB-OprA and BpeAB-OprB efflux pumps

AmrAB-OprA, the major efflux pump mediating aminoglycoside resistance in *Burkholderia pseudomallei*, is closely related to the MexXY efflux pump in *Pseudomonas aeruginosa* (Podnecky et al., 2015). Likewise, the BpeAB-OprB efflux pump in *B. pseudomallei*, a homolog of the MexAB-OprM efflux pump in *P. aeruginosa*, is not only associated with aminoglycoside and macrolide resistance but also required for the extracellular transportation of AIs (Chan and Chua, 2005; Chan et al., 2007). A significant reduction of biofilm formation in *B. pseudomallei* has been observed in the *bpeAB* deletion mutant (Chan and Chua, 2005). Additionally, the lack of extracellular AIs has been detected in the *bpeAB* deletion mutant (Chan and Chua, 2005). Later the author further demonstrated that BpeAB-OprB is responsible for the extracellular efflux of AIs (Chan et al., 2007). Therefore, the deletion of the BpeAB-OprB efflux pump attenuated biofilm formation and relative virulence by blocking AIs (Chan and Chua, 2005; Chan et al., 2007).

In contrast, Mima and Schweizer (2010) found no change of biofilm formation in the mutant only lacking BpeAB-OprB but significantly decreased biofilm formation in the mutants lacking only AmrAB-OprA or both AmrAB-OprA and BpeAB-OprB in *B. pseudomallei*. Although the exact pathway remains unclear, this study confirmed that the alteration of biofilm formation was not associated with AHLs extruding or swimming motility. So far, there has been little agreement on the role of BpeAB-OprB in biofilm formation.

Apart from the efflux pumps described above, increasing novel RND efflux pumps with positive effects on biofilm formation have been identified. For example, a recent study characterized the function of the gene *a1s_0116* encoding an RND-type transporter in biofilm formation of *A. baumannii* (Blaschke et al., 2021). In this study, the lack of *a1s_0116* resulted in deficient surface motility and pellicle biofilm formation (Blaschke et al., 2021).

#### 3.1.2 MFS-type efflux pumps

The MFS superfamily, one of the largest transporter families, possesses the most diverse transporters with a broad range of substrates including metabolites, amino acids, peptides, nucleosides, ions, and antibiotics (Saier et al., 2016). NorA, belonging to the MFS superfamily, is the major efflux pump conferring intrinsic ciprofloxacin resistance to *Staphylococcus aureus* (Papkou et al., 2020). A recent study revealed that the *norA* overexpression mutant enhanced biofilm formation in *Staphylococcus pseudintermedius* by the upregulation of *icaA* (Rampacci et al., 2022). The *icaA* gene encodes the polysaccharide intercellular adhesin forming the extracellular matrix of staphylococcal biofilm. This work indicates the positive regulatory effect of NorA on biofilm formation by regulating biofilm-associated gene expression. Furthermore, the elevated biofilm formation in this study was abolished by EPIs such as thioridazine and reserpine confirming the role of efflux pumps in biofilm formation. Another member of the MFS superfamily, the Bcr/CflA efflux system is mainly responsible for bicyclomycin, florfenicol, and chloramphenicol resistance (Smith et al., 2009). It has been reported that the disruption of *bcr* resulted in compromised biofilm formation in *Proteus mirabilis* by decreasing swimming and swarming motilities (Holling et al., 2014).

The EmrAB efflux system confers antimicrobial resistance such as nalidixic acid, thiolactomycin, and nitroxoline to *Salmonella* (Nishino et al., 2006) and *E. coli* (Puértolas-Balint et al., 2020), and contributes to colistin resistance in *A. baumannii* (Lin et al., 2017). In line with previous results in *Salmonella* (Baugh et al., 2012), the lack of *emrA*/*emrB* genes also led to diminished biofilm formation in *A. baumannii* (Lin et al., 2020). Furthermore, an array of MFS-type efflux pumps such as EmrD, EmrK, and Rv1877 in *E. coli* (Matsumura et al., 2011; Adhikary et al., 2022) and AbaF in *A. baumannii* (Sharma et al., 2017) have also been characterized to positively regulate biofilm formation. However, our understanding of how these efflux pumps mediate biofilm formation is notably underdeveloped.

#### 3.1.3 ABC-type efflux pumps

The ABC-type efflux pumps are found in all living organisms and are able to extrude diverse substances such as metabolites, vitamins, amino acids, lipids, peptides, ions, and antibiotics. The MacAB-TolC efflux system (ABC-type) is the major contributor to macrolide resistance in several species including *E. coli*, *S. maltophilia*, *K. pneumoniae*, and *A. baumannii* (Kobayashi et al., 2001; Fitzpatrick et al., 2017; Okada et al., 2017). Similar to the structure of the AcrAB–TolC efflux system mentioned previously, the MacAB-TolC efflux system is also a tripartite complex where MacB acts as an atypical ABC-type transporter, and MacA is an adaptor protein (Okada et al., 2017). Robin et al. (2021) observed the overproduction of the MacAB-TolC efflux pump in the solid–liquid biofilm of *A. baumannii*. Consistently, the deletion of *macAB-tolC* decreased the biofilm formation by 33% which accords with earlier findings in *Salmonella* (Baugh et al., 2012). A variety of physiological alterations including reduced cell metabolic activities in biofilm, lower tolerance to envelope stress, higher membrane fluidity, and downregulation of proteins involving the iron and sulfur homeostasis have been observed following the deletion of *macAB-tolC* (Robin et al., 2021). The results suggest the important role of the MacAB-TolC efflux pump in the maintenance of membrane rigidity, tolerance to high osmolarity conditions, and iron and sulfur homeostasis. Thus, MacAB-TolC might promote biofilm formation during the mature phase by providing osmotic protection and maintenance of iron homeostasis.

#### 3.1.4 Other efflux families

The SMR-type efflux pumps mediate the resistance to both antibiotics and commonly used antiseptics (Bay et al., 2008), while the MATE-type efflux pumps are the major contributor to the resistance towards fluoroquinolones and aminoglycosides (Hvorup et al., 2003). Compared with the efflux pumps of other types, the members from the SMR and MATE families are less identified to have regulatory effects on biofilm formation. As mentioned above, the deletion of *emrE*, a representative member of the SMR family, resulted in a significant reduction of biofilm formation in *E. coli* (Matsumura et al., 2011). Likewise, the absence of *mdtK*, a MATE-type efflux gene associated with resistance to norfloxacin, led to impaired biofilm formation in *Salmonella* (Nishino et al., 2006; Baugh et al., 2012). Nevertheless, the underlying mechanisms by which these efflux pumps mediate biofilm formation are poorly understood. The PACE family is the most recently discovered family with the identification of its first member, AceI in 2015 (Hassan et al., 2015). The PACE family plays a significant role in biocide resistance such as benzalkonium and chlorhexidine in Gram-negative bacteria (Hassan et al., 2018). However, little is known regarding the association between the PACE-type efflux pumps and biofilm formation.

#### 3.1.5 TolC

**Figure 2:**
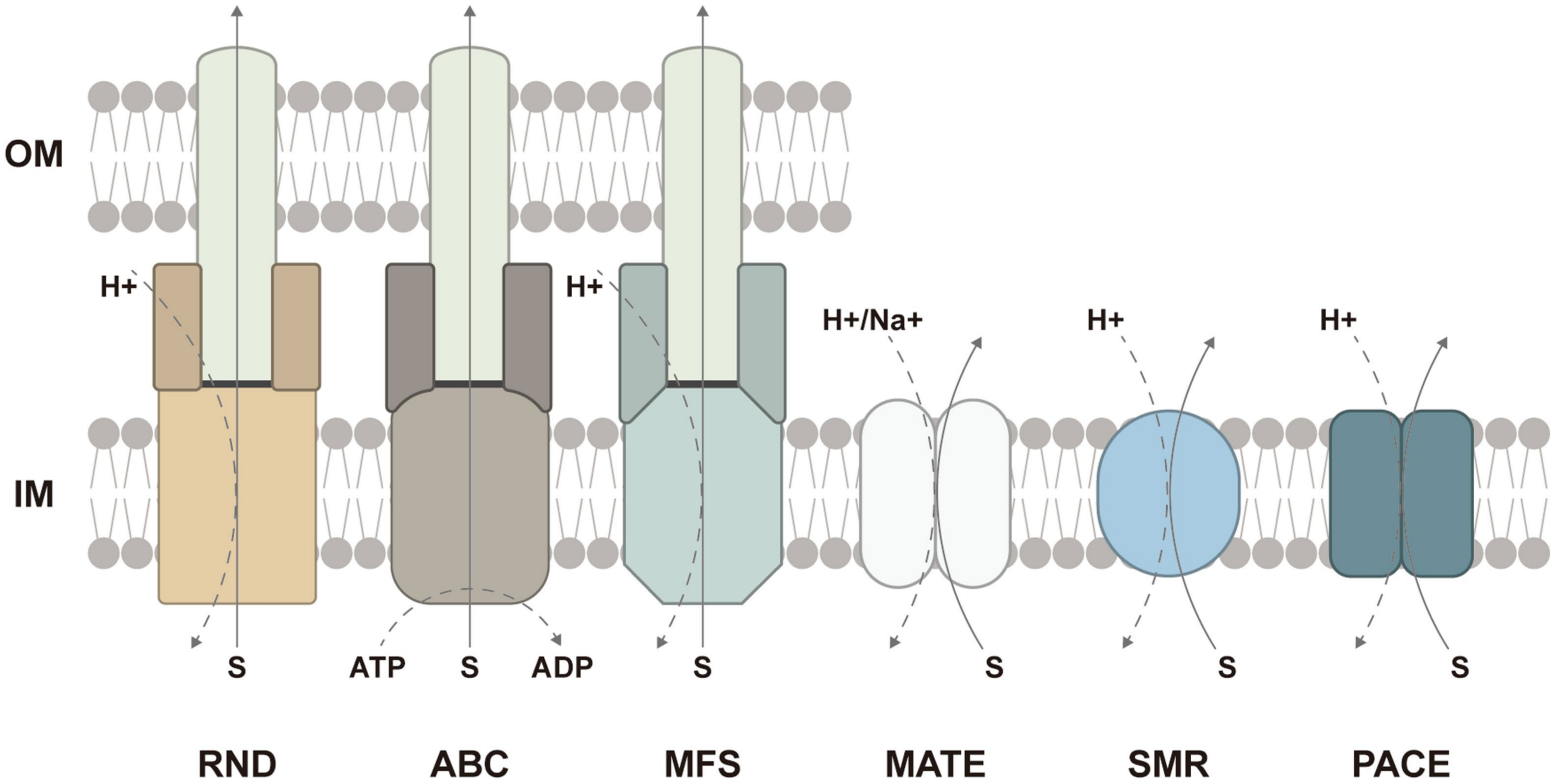
Schematic presentation of six families or superfamilies of multidrug efflux pumps and the energy sources: the resistance-nodulation–cell-division (RND) superfamily, the ATP-binding cassette (ABC) superfamily, the major facilitator superfamily (MFS), the multidrug and toxic compound extrusion (MATE) family, the small multidrug resistance (SMR) family, and the proteobacterial antimicrobial compound efflux (PACE) family. The ABC-type efflux pumps are energized by ATP hydrolysis, while the efflux pumps from other families are driven by the proton motive force. In Gram-negative bacteria, the RND-, ABC-, and MFS-type efflux pumps frequently form tripartite complexes comprised of inner membrane pumps, periplasmic adapter proteins, and the outer-membrane protein channel to span both the inner and outer membranes. The ABC-and MFS-type efflux pumps can also function in single-component forms. IM, inner membrane; OM, outer membrane.

In addition to multiple efflux pumps, several studies have revealed that the loss of TolC also contributed to biofilm inhibition. TolC is an outer membrane protein channel binding to efflux pumps such as MdtABC and AcrAB (RND-type), EmrAB (MFS-type), and MacAB (ABC-type) in Gram-negative bacteria (Koronakis et al., 2004; Greene et al., 2018). In different tripartite complexes, the structure and function of TolC are similar whereas inner membrane pumps are responsible for the determination of substrate specificity (Zgurskaya, 2009; Yousefian et al., 2021) (Figure 2). Afterward, periplasmic adapter proteins accommodate the diversity to facilitate interactions between inner membrane pumps and TolC. Finally, the delivered substrates are transported passively through the outer membrane channel.

It has been well established that the loss of *tolC* compromises biofilm formation especially in *E. coli* (Hou et al., 2014; Bay et al., 2017; Li et al., 2018; Wang et al., 2021). The loss of TolC could downregulate curli fimbriae biosynthesis thereby abolishing the aggregation and adherence during the initial stage of biofilm formation (Hou et al., 2014; Li et al., 2018). Nevertheless, owing to the pleiotropic functions of TolC involving diverse efflux pumps such as EmrAB, AcrAB, AcrEF, and AcrAD in *E. coli* (Nikaido and Takatsuka, 2009), existing research failed to discern which efflux pump was involved in mediating biofilm formation. Imuta et al. (2008) demonstrated the role of TolC in biofilm formation of Enteroaggregative *Escherichia coli*. In this study, the *tolC* deletion mutant exhibited diminished biofilm formation due to the loss of the bacterial surface hydrophobicity and decreased expression of aggregative fimbriae. Even though this study ruled out the involvement of AcrA in mediating biofilm formation, whether other efflux systems participated was not investigated. Future studies are needed to decipher the exact mechanisms.

On the other hand, as a consequence of the pleiotropic functions, the loss of *tolC* caused the most significant reduction of biofilm formation in *E. coli* compared with the individual deletion of a variety of efflux pumps (Bay et al., 2017). Moreover, controversial outcomes were obtained in different growth media. Significantly decreased biofilm formation caused by the loss of *tolC* was only found in rich media in contrast to the elevated biofilm formation in minimal media (Bay et al., 2017). These results reflect the divergence in efflux pump functions derived from distinct growth conditions.

In addition to *E. coli*, the absence of TolC also resulted in alterations of surface hydrophobicity and significantly attenuated aggregation leading to the compromised biofilm formation in *Actinobacillus pleuropneumoniae*, which was restored by the gene complementation of *tolC1* (Li et al., 2016a). Moreover, a significant downregulation of the poly-β-1, 6-N-acetyl-D-glucosamine (PGA), an important biofilm matrix component of *A. pleuropneumoniae* was revealed in the *ΔtolC* mutant by confocal microscopy and quantitative RT-PCR (Li et al., 2016b).

### 3.2 Negative effects

On the other aspect, many studies have also revealed the negative effects of efflux pumps on biofilm formation by reducing AIs and mediating relative gene expression. Bay et al. (2017) investigated the impact of single deletion of various efflux pump genes belonging to different families (RND: *acrA*, *acrB*, *acrD*, *acrE*, and *acrF*; MATE: *mdtK*; MFS: *emrA* and *emrB*; and SMR: *emrE*, *sugE*, *mdtI*, and *mdtJ*) on biofilm formation in *E. coli*. The results showed that all deletion mutants exhibited similar or enhanced biofilm formation compared with the wild-type strain in contrast to previous findings in *Salmonella* (Baugh et al., 2012), *E. coli* (Matsumura et al., 2011), and *A. baumannii* (Lin et al., 2020). Although it is difficult to explain this outcome, the difference might partly be attributed to different methodologies such as growth medium and plates. Previous research predominantly quantified biofilm formation by the crystal violet staining method. The major drawback of this approach is the lack of reproducibility. The results differ remarkably from biofilm growth conditions, solvent concentrations, and the decolorization time (Pantanella et al., 2013). Furthermore, the study conducted by Matsumura et al. (2011) demonstrated that more biofilm was built in hydrophilic polystyrene plates compared with hydrophobic ones, which are commonly used for biofilm formation evaluation. In the work of Bay et al. (2017), biofilms are cultivated in peg lids of the Calgary Biofilm Device. Another possible explanation is that some efflux pumps can export substrates required for biofilm formation with unique specificity. Thus, the loss of such efflux pumps might enhance biofilm formation by hindering the expelling of essential substances. Future work is needed to unravel the exact substance extruded by efflux pumps and underlying pathways. Moreover, this study highlights that some efflux pumps mediated biofilm formation in a time-dependent manner. For example, the lack of *acrE* (RND-type); *mdtK* (MATE-type); *emrA* and *emrB* (MFS-type); *emrE* and *sugE* (SMR-type) only impacted the early stage of biofilm formation.

The MexEF-OprN efflux pump, belonging to the RND family, is responsible for transporting fluoroquinolones, chloramphenicol, and trimethoprim (Juarez et al., 2018). *P. aeruginosa* mutants overexpressing *mexEF-oprN* displayed impaired swarming motility and biofilm formation by reducing AHLs (Favre-Bonté et al., 2003; Lamarche and Déziel, 2011). Lamarche and Déziel (2011) found the extracellular accumulation of 4-hydroxy-2-heptylquinoline (HHQ) in the cultures and the intracellular insufficiency of HHQ in the mutants overexpressing *mexEF-oprN*. In addition, the synthesis of the Pseudomonas Quinolone Signal (PQS) was greatly compromised (Lamarche and Déziel, 2011). HHQ is the precursor of PQS, a main AI of QS systems in *P. aeruginosa*. These findings indicate that the failure of PQS synthesis was due to the rapid exportation of HHQ out of cells by the MexEF-OprN efflux pump. The capacity of MexEF-OprN to extrude HHQ was further confirmed by finding that the inactivation of MexEF-OprN using an EPI increased intracellular concentrations of HHQ and decreased extracellular concentrations of HHQ in supernatants. Consistently, the inactivation of MexEF-Opr by the EPI or the deletion of *mexE* encoding the MexEF-OprN efflux pump restored PQS syntheses and biofilm formation (Lamarche and Déziel, 2011). Taken together, the evidence suggests the negative effect of MexEF-OprN on biofilm formation by the disruption of AI synthesis.

In addition to the inhibitory effect of *adeB* deletion on biofilm formation in *A. baumannii*, Yoon et al. (2015) also revealed the negative effects of AdeABC and AdeIJK efflux pumps on biofilm formation by impacting the initial attachment. The diminished biofilm formation in *A. baumannii* has been observed in *adeABC* and *adeIJK* overexpressing mutants (Yoon et al., 2015). In these mutants, the downregulation of CsuA/B and CsuC, and the FimA fimbrial proteins responsible for initial adherence, surface colonization, and microcolony formation during the early stage of biofilm formation has been found by the proteomic analysis (Sivaneson et al., 2011). Furthermore, the expression of the diaminobutyrate-2-oxoglutarate aminotransferase (DABA-AT) was also downregulated in the mutants overexpressing *adeABC* and *adeIJK*. DABA-AT plays a role in the biosynthesis of DAP, a polyamine required for *A. baumannii* surface-associated motility (Skiebe et al., 2012). These results collectively suggest the impact of AdeABC and AdeIJK on the early stage of biofilm formation in *A. baumannii*.

Furthermore, some efflux pumps negatively regulate biofilm formation by modulating the relative gene expression. Zhu et al. (2008) characterized the role of the lm. G_1771 gene encoding a novel ABC-type transporter in *Listeria monocytogenes* as a negative regulator of biofilm formation. In 2011, the same team (Zhu et al., 2011) further illuminated the function of the lm. G_1771 gene. The transcriptomics analysis demonstrated that this gene involved the biofilm formation signal pathway by regulating the genes encoding cell surface proteins, cell surface anchor proteins, and transcriptional regulators.

## 4 The effects of EPIs on biofilm formation

EPIs are substances with properties of blocking efflux pump activities. In the past decades, there has been a surge of interest in searching for EPIs to reverse antibiotic resistance and inhibit biofilm formation (Fiamegos et al., 2011; Kalia et al., 2012). EPIs can be classified by distinct action modes such as repression of efflux genes, remodeling substrates, blocking active efflux pump assembly, and inhibiting uptake of efflux pumps. Within this category, carbonyl cyanide m-chlorophenylhydrazone (CCCP), 1-(1-napthylmethyl)-piperazine (NMP), and PAβN are the most extensively studied EPIs with precise action modes. CCCP is an ionophore with the capacity to block proton motive force leading to the inhibition of RND-type efflux pumps. Given this action mode, CCCP not only inhibits the activities of efflux pumps but also influences the metabolite activity of bacteria. Therefore, it remains disputable that the effects of CCCP on biofilm formation or antibiotic resistance derive from efflux pump inhibition or the influence on metabolite. PAβN and NMP are also broad-spectrum inhibitors of RND efflux pumps, but act by directly binding to efflux pumps such as the AcrAB-TolC efflux system in *E. coli* (Chevalier et al., 2004) and the MexAB-OprM efflux system in *P. aeruginosa* (Renau et al., 1999). Good inhibitory effects of PAβN and CCCP on biofilm formation have been revealed in many species such as *A. pleuropneumoniae* (Li et al., 2016a), *Salmonella* (Baugh et al., 2012), *E. coli* (Kvist et al., 2008; Baugh et al., 2014), *K. pneumoniae* (Kvist et al., 2008), *P. aeruginosa* (Liu et al., 2010; Baugh et al., 2014), *S. aureus* (Kvist et al., 2008; Baugh et al., 2014), and *Pseudomonas putida* (Kvist et al., 2008). Recently, a study also demonstrated the effectiveness of PAβN on the inhibition of biofilm formation in Carbapenem-resistant *P. aeruginosa* (CRPA) clinical isolates (Li et al., 2022). However, the single application of NMP showed relatively poor activity on biofilm inhibition (Kvist et al., 2008). Both PAβN and NMP possess toxicity to mammalian cells rendering challenges to their clinical use.

As the action modes of many EPIs remain unclear, they can also be categorized into nature and synthesis according to their origins (Sharma et al., 2021). Several natural EPIs originating from plants have been characterized to contribute to biofilm control. For example, baicalin, a natural flavonoid compound extracted from roots of *Scutellaria baicalensis*, possesses antibacterial, antiviral, and immune-enhancing activities (Moore et al., 2016). It has been demonstrated that baicalin reduced biofilm formation in *Staphylococcus saprophyticus* by inhibiting the MsrA efflux pump (ABC-type) (Wang et al., 2019). 4′,5′-O-dicaffeoylquinic acid (4′,5′-ODCQA), a plant EPI separated from *Artemisia absinthium*, was able to diminish biofilm formation in *S. aureus* and *Enterococcus faecalis* by inhibiting MFS-type efflux pumps (Fiamegos et al., 2011). 4-allyl-2-methoxyphenol (eugenol) is a natural phenolic bioactive component extracted from clove oil, nutmeg, and basil (Ferreira et al., 2017). Sen et al. (2023) investigated the impact of eugenol on inhibiting efflux pumps and biofilm formation in 10 azole-resistant *Aspergillus fumigatus* (ARAF) isolates collected from 245 *Aspergillus* environmental samples. The confocal laser scanning microscopy results showed that the eugenol treatment caused a loss of extracellular matrix of ARAF biofilm. Additionally, the expression of MFS-type efflux genes such as *MDR1*, *MDR4*, *erg11A*, and *MedA* was downregulated following the eugenol treatment compared with untreated isolates. Xanthone, a heterocyclic polyphenolic molecule, possesses both natural derivatives originating from higher plants, lichens, fungi, and marine products and synthesis derivatives (Pinto et al., 2021). In addition to multiple activities including antitumor, anticoagulant, antiplatelet, anti-inflammatory, antimalarial, antimicrobial, hepatoprotective, and antioxidant, the biofilm inhibitory effect of xanthones has also been characterized recently (Durães et al., 2021). However, the synthesis of natural EPIs remains a main obstacle due to the complex and bulky structure.

In order to circumvent potential problems of searching for novel EPIs, there have been attempts to select EPIs from available drugs. For example, thioridazine, an antipsychotic drug, is able to inhibit the MFS-type efflux pumps in *S. aureus* and the RND-type efflux pumps in *A. baumannii* (Kaatz et al., 2003; Piddock, 2006; Kvist et al., 2008; Ahmadi et al., 2022). Nzakizwanayo et al. (2017) assessed the potential of fluoxetine and thioridazine as EPIs to control crystalline biofilm formation in *P. mirabilis* associated with the Bcr/CflA efflux system (MFS-type). Both EPIs significantly decreased crystalline biofilm formation on catheters in *P. mirabilis* by reducing swimming and swarming motilities. In addition to *P. mirabilis*, significantly decreased biofilm formation caused by thioridazine has also been observed in other species including *P. aeruginosa*, *E. coli*, *K. pneumoniae*, *S. aureus*, and *P. putida* (Kvist et al., 2008; Nzakizwanayo et al., 2017). Especially the combined application of thioridazine and PAβN showed remarkable biofilm inhibitory effects (up to 99% reduction) (Kvist et al., 2008). In Zimmermann et al. (2019), reported that the first approved drug, nilotinib, a tyrosine kinase inhibitor, significantly abolished the biofilm formation combined with ciprofloxacin at clinically viable concentrations by inhibiting the NorA efflux pump (MFS-type).

Although the effectiveness of EPIs on biofilm inhibition has been demonstrated in many species, there are still several hurdles on the way to clinical use of EPIs as a promising biofilm inhibition strategy. From an economic perspective, improving antimicrobials in use is more attractive than developing a new chemical entity for pharmaceutical companies (Lomovskaya and Bostian, 2006). Additionally, the toxicity issue of EPIs is a great challenge for clinical use. The potential strategies include the combination of EPIs and antimicrobial compounds, local utilization, and application of EPIs on medical devices (Dawan et al., 2022). Furthermore, earlier studies have indicated that the definitive biofilm formation is a synergistic result of multiple efflux pumps. Once one of the efflux pumps is inhibited, the expression of other efflux pumps can be upregulated for compensation by a feedback mechanism (Blair et al., 2015; Liu et al., 2017). Thus, it is estimated that the combination of diverse EPIs renders better activity (Kvist et al., 2008). Future investigations on the combination of diverse EPIs or EPIs and other antimicrobial agents are recommended.

## 5 Conclusion

A good understanding of efflux pump functions in biofilm formation is of great importance for selecting EPI targets. Although the regulatory effects of many efflux pumps on biofilm formation have been characterized, the precise mechanisms by which these efflux pumps mediate biofilm formation are not fully understood. Especially the role of some efflux systems such as Acr, Ade, and EmrAB in biofilm formation is still controversial. Additionally, previous research suggests that the expression of efflux pumps in biofilm formation differs among strains, species, and growth conditions such as media and antimicrobial selective pressure. The question about how condition factors influence the expression of efflux pumps remains unanswered. Furthermore, what is known about the effects of efflux pumps on biofilm formation is largely based on efflux mutants using standard strains. There is a paucity of relative information on clinical strains. Future efforts are needed to fully clarify these problems.

## Author contributions

JR: Writing – original draft. MW: Software, Writing – review & editing. WZ: Conceptualization, Writing – review & editing. ZL: Writing – review & editing.
